# Social and Independence Skills Dynamics in Adolescents with Developmental Disorders Comprehensive Intervention Program Results

**DOI:** 10.1192/j.eurpsy.2025.2179

**Published:** 2025-08-26

**Authors:** A. Portnova, N. Maltseva, K. Reitsen

**Affiliations:** 1 Department of Psychiatry, Psychotherapy and Psychosomatic Pathology, Patrice Lumumba Peoples’ Friendship University of Russia; 2 Association of Psychiatrists and Psychologists for Evidence-Based Practice; 3 Center for Curative Pedagogics, Moscow, Russian Federation

## Abstract

**Introduction:**

Adolescents with developmental disabilities and their families face significant challenges in the transition to adulthood. Comprehensive interventions that include psychological support, daily living skills training, and vocational guidance are crucial. This pilot study tests a program aimed at developing independence and adaptive skills in adolescents with developmental disorders.

**Objectives:**

The study aimed to evaluate the dynamics of adaptive and independence skills in adolescents with various developmental disorders who participated in a comprehensive intervention program.

**Methods:**

10 adolescents (5 boys and 5 girls, mean age 15,11, SD 2,4) were included in the study. Participants were mainly diagnosed with the following primary DS: F70.xx, F84.xx. Also, participants had additional DS such as F48.xx, F80.xx, G40.xx, Q37.xx, Q74.xx. IQ of the participants was measured by the Leiter-3 Performance Scales (mean 62.8, SD 26.9). Comprehensive intervention program lasted the 2022/2023 academic year 3 d/week, 2-3 h/day. The intervention included individual and group sessions, several home visits and the parent groups led by a team of psychoeducational professionals (neuropsychologists, special educators, speech pathologists). The training outcomes were measured by the Vineland Adaptive Behavior Scales (VABS-2). Statistical analysis was performed using the paired samples t-tests and d-Cohen effect size (d).

**Results:**

Significant improvements were observed in all four VABS subscales. Communication improved from 56.1 to 59.6 (t=-3.42, p=0.008, d=-1.08), Daily Living Skills from 59.9 to 66.4 (t=-5.57, p<0.001, d=-1.76), Socialisation from 58.6 to 65.1 (t=-3.84, p=0.004, d=-1.21), and General Adaptive Behaviour Index from 56.9 to 62.0 (t=-5.31, p<0.001, d=-1.68). The largest improvements were seen in Daily Living Skills and General Adaptive Behaviour Index which highlight the program’s effectiveness in fostering independence and adaptive capacities in adolescents.

**Conclusions:**

The pilot study demonstrated the promising effectiveness of the program in developing independence and adaptive skills, suggesting it as a valuable intervention for preparing young people for independent living in adulthood. Following research plans include follow-up analysis of current participants’ outcomes, an increase in sample size, and the implementation of between-group designs.

**Disclosure of Interest:**

None Declared

